# Mixing Optimization in Grooved Serpentine Microchannels

**DOI:** 10.3390/mi11010061

**Published:** 2020-01-04

**Authors:** Tyler Rhoades, Chandrasekhar R. Kothapalli, Petru S. Fodor

**Affiliations:** 1 Department of Physics, Cleveland State University, 2121 Euclid Avenue, Cleveland, OH 44236, USA; t.d.rhoades@vikes.csuohio.edu; 2 Department of Chemical and Biomedical Engineering, Cleveland State University, 2121 Euclid Avenue, Cleveland, OH 44236, USA; c.kothapalli@csuohio.edu

**Keywords:** passive micromixers, Dean flows and mixers, serpentine channels, staggered herring bone (SHB) mixers, mixing index

## Abstract

Computational fluid dynamics modeling at Reynolds numbers ranging from 10 to 100 was used to characterize the performance of a new type of micromixer employing a serpentine channel with a grooved surface. The new topology exploits the overlap between the typical Dean flows present in curved channels due to the centrifugal forces experienced by the fluids, and the helical flows induced by slanted groove-ridge patterns with respect to the direction of the flow. The resulting flows are complex, with multiple vortices and saddle points, leading to enhanced mixing across the section of the channel. The optimization of the mixers with respect to the inner radius of curvature (*R_in_*) of the serpentine channel identifies the designs in which the mixing index quality is both high (*M* > 0.95) and independent of the Reynolds number across all the values investigated.

## 1. Introduction

Microfluidic devices and lab-on-a-chip systems are widely used nowadays in chemical and biological sciences [1,2,3,4] for applications ranging from the synthesis of nanoparticles and colloidal systems [5,6,7] to molecular diagnostics [8,9] and cell biology [10,11], owing to their reduced consumption of reactants [12], better control over reaction variables such as the reactant concentration and temperature [13,14], and the ability to spatially control liquid composition with cellular resolution [11,15]. Virtually any use of these devices in chemical analysis and fabrication or biological assays/bioengineering requires the ability to mix well and predictably two or more chemical or biological components [16,17]. Thus, mixing components is one of the fundamental and critical blocks in the development of microfluidic devices. It is also one of the difficult functionalities to achieve, as the fluid flow is laminar, characterized by low Reynolds numbers at the length scales involved, and the turbulent mixing techniques employed in macroscale systems are not typically applicable [18].

The challenge of achieving efficient mixing on the microscale has sustained a broad body of research on the topic. The strategies used to promote mixing in diffusion-dominated, microscale, laminar flow regimes are classified as either active or passive [17,19,20]. Active strategies integrate external drivers in the mixer design such as moving membranes [21,22], rotating magnetic particles [23], and electromagnetic [24,25] or acoustic [26,27,28] fields, in an effort to stir and mix the inhomogeneous fluid solution. While these strategies have shown good mixing capabilities, this has come at the price of more complex designs that are harder to integrate into analysis systems, and more costly to scale-up. Passive strategies, on the other hand, rely on the channel geometry–fluid interaction to induce transversal flows that stretch the interface between the fluids components to be mixed, and thus increase the efficiency of diffusional mixing, as well as promoting chaotic advection, mimicking macroscale turbulence [17,18,19]. Arrays of obstacles [28,29,30,31], ridge-groove systems [32,33,34], two- or three-dimensional turns [35,36], and curved channel sections [17,37,38] have been successfully used to exploit the already existent pressure differentials necessary to push the fluids of interest along the microfluidic device, in order to generate cross-sectional flows capable of stretching, folding, and splitting fluid elements conducive to efficient mixing. Aside from a proven ability to reach the necessary mixing quality, passive designs benefit from being accessible to a variety of fabrication methods including soft lithography [39,40], 3D printing [41], and molecular imprinting [42]. This, together with accurate computational modeling of the fluid and reactant dynamics in these devices accessible through a variety of popular CFD packages, has enabled an efficient pipeline for the design, optimization, prototyping, and testing of passive mixers [33,43,44,45].

**Figure 1:**
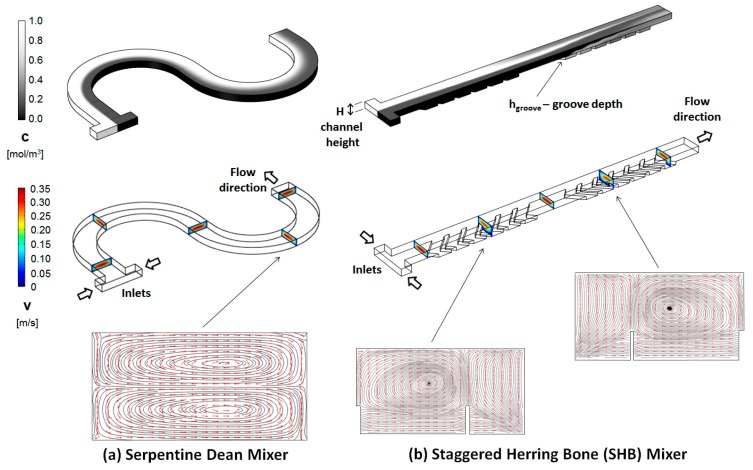
(**a**) Serpentine and (**b**) staggered herring bone (SHB) micromixer geometrical topologies. (**Top**) Concentration distribution along the channel, with the fluids to be mixed introduced through two opposite inlets; (**middle**) Geometry of the channels and the velocity field cross-sections at different positions along the channel; (**bottom**) Transversal streamline/arrow plots (*Re* = 20 for both designs).

The mixer geometrical design investigated in this work combines two of the above distinct passive strategies that have proved successful in inducing cross-sectional flows in microchannels. In the first geometrical topology (Figure 1a), a serpentine channel is used to guide the fluid flow along curved trajectories. The centrifugal forces that are experienced by the fluid lead to the formation of transversal vortices [17], which can be exploited to intermix the fluid components of interest. At Reynolds numbers below 100, two vertically stacked vortices are formed, although more complex flow geometries can be achieved by operating at higher Reynolds numbers [17], or by using geometrical modifications such as surface patterns [46], modulation of the side walls [47], misaligned inlets [48], unbalanced sub-channels [49], non-rectangular cross-sections [50], or three-dimensional turns [51]. The second geometrical topology (Figure 1b) uses an array of slanted V-shaped asymmetric groove-ridge patterns placed on the bottom [32,40], or top and bottom [48] of the channel. The resistance profile presented by the groove-ridge patterns to the fluid flow allows for the axial pressure gradient between the inlet and the outlet to drive the transversal components of the flow, resulting in horizontally stacked vortices. Shifting the apex of the ridges, in a geometry referred to as the staggered herring bone (SHB), has proved to be an efficient strategy to enhance mixing in these type of channels [52,53].

The current study takes advantage of the fact that for the two distinct topologies mentioned above, the pair of transversal vortices induced are staggered along orthogonal directions, horizontal for the serpentine channels, and vertical for the SHB ones. To that effect, a new design is proposed (termed as grooved serpentine micromixer design) in which asymmetric grooves are mapped on the curved channels and the geometries are optimized with respect to the inner radius (*R_in_*) of the curved mixing sections, and thus the number of grooves per section, to promote increased complexity in the cross-sectional flows and mixing.

## 2. Mixer Geometrical Design

**Figure 2:**
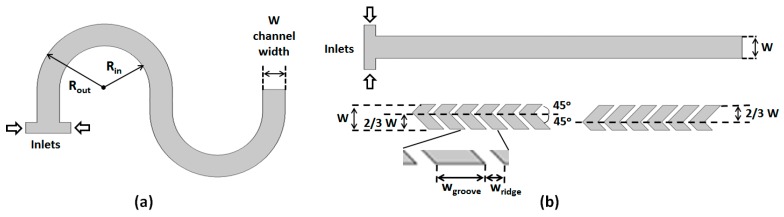
(**a**) Top view of the curved channel; (**b**) (**top**) Top view of the SHB channel; and (**bottom**) configuration of the asymmetric grooves placed on the bottom of the channel.

Figure 2 shows the basic topologies and parameters for the two micromixer geometries that are combined. The serpentine design (Figure 2a) consists of a rectangular cross-section channel (*W*—width; *H*—height) with repeating S-shaped mixing units, each formed by two semicircular sub-sections. The inner radius of curvature of each section is *R_in_*, while the outer radius of curvature is *R_out_* (= *R_in_ + W*). The two inlets through which the fluids to be mixed are introduced have the same height but half the width of the main channel. The SHB design (Figure 2b) consists of straight mixing sections with the same width, height, and length as the ones for the serpentine design. The bottom of the channel is lined up with asymmetric grooves for which the apex position switches from one side of the channel to the other, midway through the mixing section. For this study, the width *W* and the height *H* for all microchannels investigated were fixed to 200 μm and 66 μm, respectively.

For the groove design, the parameters used correspond to the optimized values for maximized mixing in SHB-type mixers as per previous studies [32,33,52,53,54,55], and are as follows: groove angle = 45° with respect to the channel longitudinal axis; apex position is at *(2/3)W* from the side of the channel; grooves of width *w_groove_* = 125 μm separated by ridges with *w_groove_* = 50 μm; and groove depths *h_groove_* = 33 μm.

**Figure 3:**
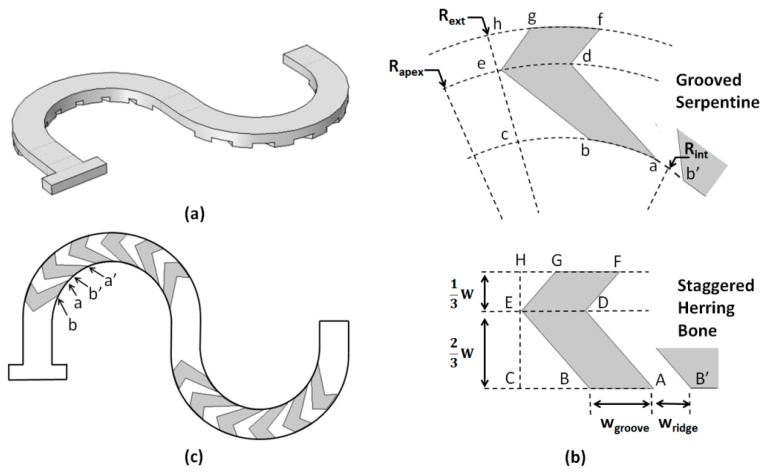
(**a**) 3D geometry of the grooved serpentine micromixer design; (**b**) Correspondence between the geometry of the grooves in the straight and curved channels; (**c**) Plane projection of the grooves on the outline of the curved channel.

To generate the new mixer topology, the SHB groove-ridge-type patterns were applied to the curved channels (Figure 3). To achieve this, firstly all the points (A, B, C, D, etc.) defining a single groove structure in the SHB straight channel geometry were mapped on the curved channel using the constraint that the resulting arc lengths *s* had the same value as their corresponding segments in the SHB (Figure 3b). Thus, *s_ab_ =* AB, *s_bc_ =* BC, *s_de_ =* DE, etc. In the radial direction, the distances of the groove apexes relative to the side walls of the channel remained the same as in the straight channel geometry, i.e., CE *= ce* = (2/3)*W* and EF = *ef* = (1/3)*W*, and thus the radius of curvature of the apex line was *R_apex_ = R_in_* + (2/3)*W*. Subsequently, the groove design was rotate-duplicated along the length of the circular mixing sub-section (Figure 3c) with the constraint that the arc length distances along the inner side channel were the same as in the original SHB design, i.e., *s_ab_ = AB = w_groove_*, *s_ab’_ = AB’ = w_ridge_, s_a’b’_ = w_groove_*, etc. Given that the length of the circular sub-section in the serpentine design was the same as the length of the straight sub-section in the SHB design, the new design had an identical number of grooves. The above procedure was repeated for the second mixing circular sub-section, resulting in a ridge-groove structure in which the apex position switched from one side to the other side of the channel relative to the direction of the fluid flow, similar with the case of the staggered herring bone configuration. For all set-ups, the depth of the grooves was kept the same as for the SHB design, i.e., *h_groove_* = 33 μm. The parameters against which the performance of the design was evaluated are the inner radius of curvature (*R_in_*) and the Reynolds number.

## 3. Numerical Modeling

To obtain the velocity profiles inside the microchannels, the Navier-Stokes equations (momentum and continuity) were solved numerically under the assumption that the fluid is incompressible and Newtonian under a steady state pressure driven flow:

(1) $$\rho \left(\frac{\partial u}{\partial t}+\left(u\cdot \nabla \right)u\right)=-\nabla p+\eta \nabla^{2}u$$

(2) ∇·u=0

where ***u*** (m∙s^−1^)] is the velocity vector, *ρ* (kg∙m^−3^) is the fluid density, *η* (kg∙m^−1^∙s^−1^) is the fluid viscosity, *t* (s) is the time, and *p* (Pa) is the pressure. The values for the density and viscosity were set to those for water at room temperature, i.e., *ρ* = 10^3^ kg·m^−3^ and *η =* 10^−3^ kg·m^−1^∙s^−1^. No-slip boundary conditions were set for the walls of the micromixers and the flow field equations were solved using a generalized minimal residual method (GMRES) iterative solver with a geometrical multigrid pre-conditioner. A free tetrahedral mesh was used for the entire microchannel, with typical mesh elements being no larger than ~6.5 μm^3^ in volume for all the models studied. For all the simulations described in this work, we used the computational package COMSOL Multiphysics 5.1 (COMSOL, Inc., Burlington, MA, USA) and its Computational Fluid Dynamics/Chemical Engineering module.

Subsequently, the flow fields obtained were used in the concentration–diffusion equation to calculate the distribution of the concentration *c* (mol∙m^−3^) through the mixer:

(3) $$\frac{\partial c}{\partial t}=D\nabla^{2}c-u\cdot \nabla c$$

where *D* (m^2^∙s^−1^) is its diffusion constant. The molar concentration *c* is assigned to be 1 (mol∙m^−3^) at one of the inlets and 0 (mol∙m^−3^) at the opposite inlet. The diffusion constant is set to 1.0 × 10^−9^ m^2^∙s^−1^_,_ corresponding to the typical diffusivity range of most ions in aqueous solutions. The same type of solver/computational package as for the flow fields modeling was used. However, the concentration–diffusion equation was solved using a finer discretization for the volume of the channels with typical meshing elements no larger than ~4 μm^3^ in volume, to avoid the errors that can be associated with modeling mass-transport phenomena.

The accuracy of the numerical models used has been validated against experimental results for both serpentine type micromixers [56,57] as well as for the staggered herring bone designs [58]. In particular, for the type of mixing measures used in this study, such as concentration maps and mixing indexes, our computational models showed good agreement with the experimental data.

## 4. Results and Discussion

In this study the proposed designs were optimized with respect to the inner radius of curvature (*R_in_*) and the average fluid speed (Reynolds number) as the primary parameters controlling the strength of the transversal flows responsible for mixing. In brief, for smaller radii of curvature (*R_in_*) and larger flow speeds, the centrifugal forces experienced by the fluids as they are pressure driven around the curved channels are larger, leading to stronger transversal Dean vortices [17,50,51]. On the other hand, a small radius of curvature geometrically limits the number of groove-ridge patterns akin to the SHB design that can be placed on the bottom of the mixing unit. If the radius of curvature is increased, more flow controlling asymmetric grooves can be placed in the fluid path, but at the expense of longer residence times, larger pressure differentials, and weaker Dean flows. In this respect, to identify the optimal design and working conditions that maximize the transversal flows resulting from the combination of Dean vortices and slanted groove driven fluid motion, the *R_in_* is parametrized with values ranging from 200 to 600 μm, corresponding to a *R_in_/W* ratio of 1 to 3, while the average inlet fluid speeds are changed from 0.1 to 1 m/s, corresponding to Reynolds numbers from 10 to 100.

**Figure 4:**
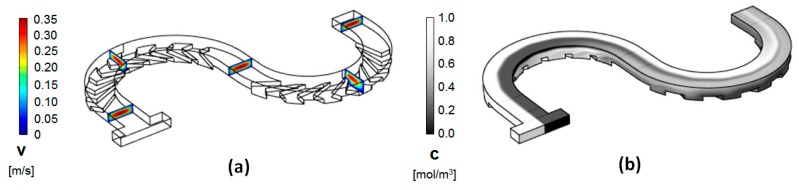
(**a**) Velocity field cross-sections at different positions along the channel; (**b**) Concentration distribution along the channel (*Re* = 20; *R_in_ =* 400 μm).

Typical results for the modeling of the flow fields and the advection and diffusion of chemical species in the new groove serpentine micromixers were shown in Figure 4. The data were collected for a design with the same channel length, radius of curvature, and flow conditions akin to the simple serpentine design shown in Figure 1. The surface concentration maps appear to be consistent with the expectations for the formation of more complex transversal flows in this type of design. In particular, in the second half of the mixing unit, rapid splitting of the fluid streams occurs, accompanied by noticeable homogenization of the concentration.

**Figure 5:**
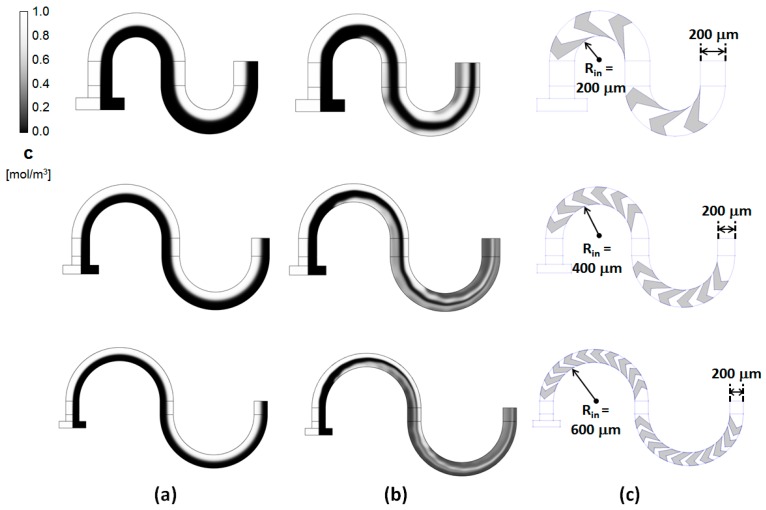
Concentration distributions along the center planes of the main channel (i.e., 33 μm below the top surface) for (**a**) simple serpentines and (**b**) grooved serpentines micromixers; (**c**) The configuration of the corresponding grooves placed on the bottom of the new designs for *R_in_* = 200 μm, 400 μm, and 600 μm, respectively.

This behavior was consistent across similar designs with different radii of curvature *R_in_* (Figure 5). The maps of the concentration evolution along the length of the serpentine channels show distinct differences between the simple rectangular channels and the grooved ones. In the simple serpentine channels, the two liquids to be mixed, entering through opposite inlets, remain distinctly separated throughout the length of the channel. While some homogenization of the concentration occurs, its extent is limited to the interface between the two fluids of interest. In contrast, in the proposed grooved micromixers, rapid lamination of the two fluid streams occurs. This starts towards the end of the first mixing sub-section and progresses rapidly as the fluid enters the second part of the mixing section. This is similar to the behavior observed in other grooved-type micromixers [32,34]. These previous studies associated the shifts between the mixing subunits in the position of the centers of rotation of the flows driven by the asymmetric grooves with a transition to complex and chaotic advection of the fluids. It should to be noted, though, that, based on the concentration maps of the grooved serpentines (Figure 5), this transition appears to set in very fast, presumably due to the overlap with the Dean type flows. The flow profiles seem to be strongly dependent on the radius of curvature and the associated increase in the number of grooves that can be added to each cycle. As the radius of curvature is increased, the lamination sets-in slightly earlier, followed by rapid striation of the fluids into thin filaments conducive to promoting the homogenization of the fluids through an increased contact area.

While the visual analysis of the concentration distribution images indicates that the proposed designs perform well at mixing the fluid streams, a more rigorous quantitative measure is needed to identify the optimal performance. To this end, transversal concentration images were collected at the outlet of each mixer. These images were procesed as 8-bit intensity images, where the maximum intensity, i.e., 255 for grayscale image data, corresponds to the maximum concentration *c* = 1 (mol∙m^−3^), and the minimum intensity, i.e., 0 for grayscale image data, corresponds to the minimum concentration *c =* 0 (mol∙m^−3^). The intensity maps were divided into equal size regions, aka bins, and the level of homogeneity in the concentration distribution was assessed, calculating the global Shannon entropy associated with the map [59]. This provides a rigorous quantitative statistical measure of the level of uniformity in the intensity concentration map through a mixing index given by:

(4) $$M=-\frac{1}{ln2}\cdot \frac{1}{N_{bins}}\cdot \overset{N_{bins}}{\underset{j=1}{\sum }}\left[c_{j}ln\left(c_{j}\right)+\left(1-c_{j}\right)ln\left(1-c_{j}\right)\right]$$

where *M* is referred to as a mixing index, *N_bins_* is the number of bins in which the image is discretized, *c_j_* is the average concentration in the bin *j* expressed as a non-dimensional number taking values between 0 (minimum concentration) and 1 (maximum concentration). The above mixing index calculation assumes that the two fluids to be mixed are incompressible. The mixing index *M* thus defined is normalized by ln(2) (where 2 corresponds to the number of components) fixing its range of values from 0 to 1. The extreme values *M* = 0 and *M* = 1 correspond to completely segregated components and perfect mixing, making this index an easy to interpret quantitative measure of the level of mixing.

**Figure 6:**
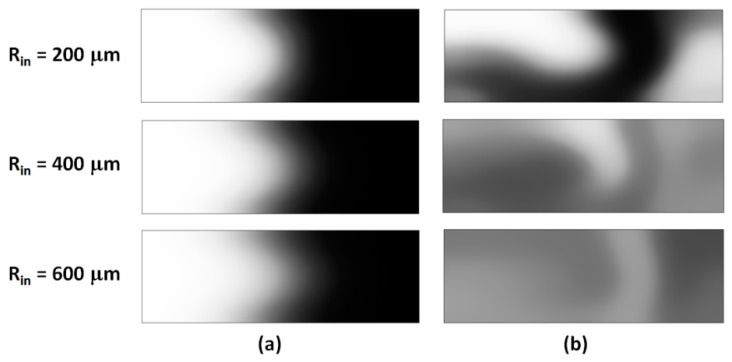
Transversal concentration distributions at the outlet of the mixing unit for (**a**) simple, and (**b**) grooved serpentines of different inner radius *R_in_* (*Re* = 10).

Intensity concentration maps at the outlet for the simple and grooved mixing sections were shown in Figure 6. As for the longitudinal cross-sections, these transversal maps show distinct differences between the two topologies. While the Dean vortices in the simple serpentines do deform the interface between the fluids, providing more surface area for the diffusion to act, the fluids remain, for the most part, in separate volumes and no global mixing is observed.

In contrast, the grooved micromixer shows a more complex distribution in the concentration maps. For *R_in_ =* 200 μm, the concentration profile (Figure 6b top) contains segregation features similar to the simple serpentine, but also exhibits lamination resulting from the overlap on the Dean flows and the flows driven by the slanted grooves. At higher radii of curvature, the contrast is even more pronounced, with a high degree of homogeneity in the distribution, and no pockets of high or low concentration regions.

**Figure 7:**
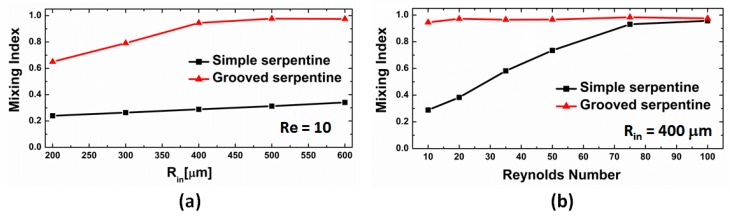
(**a**) Dependence of the mixing index on the radius of curvature *R_in_* at *Re* = 10; (**b**) Dependence of the mixing index on the Reynolds number for the design with *R_in_ =* 400 μm.

The quantitative assessment of the mixing indexes in the two designs also demonstrates that the grooved micromixers are superior in performance to the simple serpentines at all radii of curvature and Reynolds numbers. At low Reynolds numbers, for the simple serpentine mixing units, the quality of the mixing is poor and depends weakly on the radius of curvature (Figure 7a). This is not unexpected, as the strength and topology of the Dean vortices formed in curved channels is strongly dependent on the fluid speeds and complex flows conducive to promoting bifurcations, and lamination of the fluid streams develops only in the high Reynolds number regime (*Re* ≈ 100). There is a small increase in the mixing index with increased *R_in_*, which is associated with a longer residence time in the channels, but *M* does not exceed a value of 0.35 for any of the simple channels at *Re* = 10. In the grooved channels, at a low Reynolds number regime, the performance is almost three-fold higher than the simple serpentines at all radii values. Moreover, for all values of *R_in_* larger than 400 μm, i.e., two times the width of the channels, the mixing index at the end of the mixing unit is equal or larger than 0.95, indicating a high degree of homogenization. In this respect, the data indicate that grooved channels with *R_in_ =* 400 μm provide close to optimum mixing performance, while keeping the total channel length at a minimum. For smaller radii of curvature, while more mixing units can be placed in the path of the flow for the same length, the performance lags compared with the optimum. For example, results for a channel with two mixing units with *R_in_ =* 200 μm, which add up to a length 13% larger than that of a single *R_in_ =* 400 μm unit, give a mixing performance which is actually 11% smaller than the optimal design.

The Reynolds number dependence of the performance of the two types of serpentine micromixers is also telling (Figure 7b). For *R_in_ >* 400 μm, the grooved serpentines provide a high mixing quality at the outlet almost independent of the flow conditions over the whole range of fluid speeds investigated. For the un-grooved designs, the performance does improve as expected with the increase in the fluid speeds, and approaches optimal mixing at *Re* = 100, but stills lags behind the performance of the grooved ones at all Reynolds numbers.

**Figure 8:**
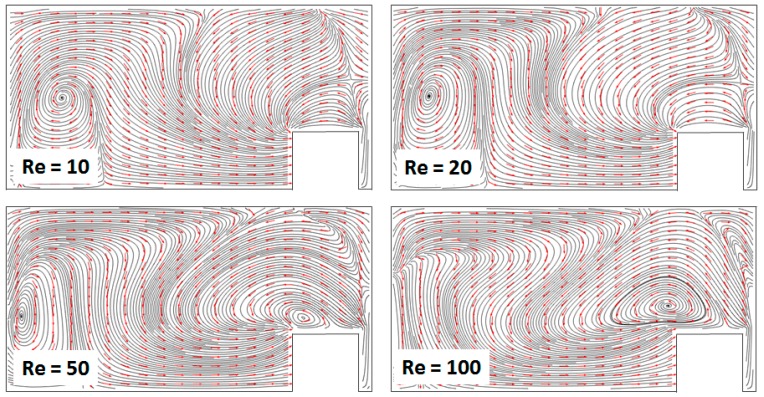
Cross-sectional streamline/arrow plots of the velocity field for the mixer with *R_in_* = 400 μm, at different Reynolds numbers. The data is collected midway through the second mixing subsection.

More insight into the topology of the flow fields that develop in these devices and drive their efficient mixing performance can be gained from reconstructing the streamline plots from the solutions to the Navier–Stokes equations (Figure 8). For all Reynolds numbers, the topology of the flows is much more complex than the one encountered in either of the basis designs, i.e., the Dean serpentine mixer and the staggered herring bone mixer. Multiple vortices were observed that extend and interact throughout the volume of the channel, resulting in the extensive folding and stretching of the fluid elements. Additionally, features that are not present in either of the original designs could be observed, such as saddle points, where parallel streamlines diverge from each other. This behavior is consistent with a transition to a chaotic advection-dominated regime, resulting in the breakup of the fluids and rapid increase in the contact area between the components to be mixed [18].

Based on the above, overlapping the Dean vortices’ structure encountered in curved channels with an orthogonal flow structure induced using slanted asymmetric grooves seems to be a very effective strategy of inducing the type of fluid motion required for efficient mixing in microchannels. Designs with *R_in_ = 2W =* 400 μm are optimal as they achieve maximized mixing using the shortest mixing unit length. Compared with simple serpentine mixers, the new designs performed three-fold better at low Reynolds numbers and remained superior throughout the range of inlet speeds investigated. Noteworthy is the fact that the performance of these mixers is almost independent of the Reynolds number, making them easy to integrate across a variety of conditions. While the fabrication of the proposed designs is slightly more complex, as it requires fabricating both the main channel structure as well as the groove structure, it is well within the capabilities of typical methods used for microfluidic device development [39]. In particular, the proposed devices can be fabricated using soft-lithography techniques [40,60] based on replica molding transfer to PDMS polymer from silicon stamps prepared using two-layer lithography. The use of oxygen plasma-activated PDMS polymer also provides high wettability for the channel surfaces [60], consistent with the assumption made in the above numerical study of no-slip boundary conditions at all the walls.

## 5. Conclusions

A new topology for a serpentine micromixer employing an asymmetric groove-ridge structure on the bottom of the channel has been evaluated numerically. At the fluid velocities used, guiding the fluid along curved channels induces vertically stacked transversal vortices, while the slanted groove-ridges with respect to the direction of the flow are associated with horizontally stacked vortices. The overlap between the two flow topologies results in very complex flow fields characterized by bifurcations and saddle points that both stretch the interface between the fluids of interest as well as promoting chaotic advection. In the range of Reynolds numbers investigated, *Re* = 10–100, in the optimized designs in which the inner radius of curvature *R_in_* is at least *2W =* 400 μm, the degree of mixing exceeds 95% and shows very little variation. Thus, one of the benefits of the proposed design is its very good consistency across different flow operation conditions.
